# The Effects of SGLT2 Inhibitors on Muscle Health in Older Adults: A Systematic Review and Meta‐Analysis

**DOI:** 10.1002/prp2.70232

**Published:** 2026-03-11

**Authors:** Weena Joongpan, Nittaya Boonmuen, Pimthong Sinchai, Sarawut Lapmanee, Natawat Klamsakul, Nutthapoom Pathomthongtaweechai

**Affiliations:** ^1^ Department of Physiology, Faculty of Science Mahidol University Bangkok Thailand; ^2^ Chulabhorn International College of Medicine Thammasat University Pathum Thani Thailand; ^3^ Department of Mathematics, Faculty of Science King Mongkut's University of Technology Thonburi Bangkok Thailand; ^4^ Chakri Naruebodindra Medical Institute, Faculty of Medicine Ramathibodi Hospital Mahidol University Samut Prakan Thailand

**Keywords:** aging, muscle mass, SGLT2 inhibitor, type 2 diabetes mellitus, weight reduction

## Abstract

Type 2 diabetes mellitus (T2DM) and obesity are growing global health concerns, particularly in older adults who are at higher risk of sarcopenia. While sodium‐glucose cotransporter 2 (SGLT2) inhibitors show promise for glycemic control and weight loss, their effects on muscle health remain unclear. We examined the effects of SGLT2 inhibitors on body weight, fat mass, and muscle mass in T2DM patients. We systematically searched the PubMed, Embase, Scopus, and Cochrane databases for relevant randomized controlled trials (RCTs). Three reviewers screened the studies, and two extracted data and assessed their quality. R software was used to evaluate heterogeneity via Cochran's *Q* and *I* ^2^ statistics. Eight RCTs (*n* = 541) were included. SGLT2 inhibitors significantly reduced body weight (standardized mean difference (SMD) = −0.85, *p* < 0.001; *I* ^2^ = 0%) and fat mass (SMD = −0.53, *p* < 0.001; *I* ^2^ = 51.1%). A small reduction in muscle mass was observed (SMD = −0.35, *p* < 0.001; *I* ^2^ = 22.9%), though substantially smaller than fat loss. Subgroup analysis confirmed that fat mass was reduced with dapagliflozin/ipragliflozin (SMD = −0.67, *p* < 0.001; *I* ^2^ = 26.4%) and empagliflozin (SMD = −0.53, *p* < 0.001; *I* ^2^ = 66.4%). SGLT2 inhibitors effectively reduce body weight primarily through fat loss in older adults. Although muscle mass declined modestly, the predominance of fat loss suggests weight reduction occurs through favorable metabolic changes. Given the slight muscle mass changes and study heterogeneity, careful monitoring in older adults is warranted, and further studies in diverse populations are needed.

## Graphical abstract

SGLT2 inhibitors reduce skeletal muscle mass modestly in older adults with type 2 diabetes. Forest plot (a) and count our funnel plot (b) show a small but significant pooled reduction in muscle mass (SMD = −0.36, 95% CI [−0.56, −0.15]; *I* ^2^ = 24.1%), with weight loss occurring predominantly through fat mass reduction.

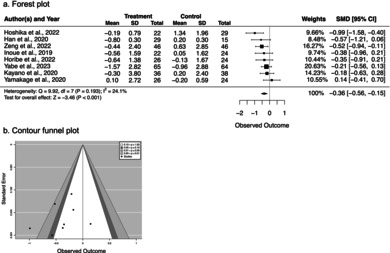

## 1 Introduction

Diabetes mellitus (DM) and obesity are major global health crises [1]. The rising obesity rate is contributing to the increasing prevalence of DM and its severe complications, which include renal, nervous, and cardiovascular diseases [2, 3, 4]. More than two billion adults are overweight or obese worldwide, and these conditions elevate the risk of developing chronic conditions, including various cancers [5]. To address these issues and their significant economic burdens, it is essential to adopt a comprehensive approach that combines prevention, the promotion of healthy lifestyles, improved access to healthcare, and the strategic use of novel classes of antidiabetic drugs, such as sodium‐glucose cotransporter 2 (SGLT2) inhibitors and glucagon‐like peptide 1 (GLP‐1) receptor agonists.

SGLT2 inhibitors (e.g., dapagliflozin, canagliflozin, and empagliflozin) represent a significant advancement in the pharmacological management of type 2 diabetes mellitus (T2DM), particularly for overweight and obese patients [6]. By selectively inhibiting SGLT2 in the renal proximal tubule, which reabsorbs 80% – 90% of filtered glucose, these drugs prevent the reabsorption of glucose from the renal filtrate back into the systemic circulation, leading to increased urinary glucose excretion or glucosuria [7, 8]. This results in a reduction in both postprandial and fasting blood glucose levels, independent of insulin secretion or hepatic glucose production, which leads to improved glycemic control and a reduced risk of hypoglycemia [9, 10, 11]. It has been shown that SGLT2 inhibitors can cause weight loss by increasing glucose excretion and improving fat metabolism [12].

In addition, SGLT2 inhibitors provide robust cardiovascular and renal protection; for example, they have been found to reduce the risk of major adverse cardiovascular events and heart failure hospitalization and slow the progression of kidney diseases [13, 14, 15, 16]. These benefits are associated with reduced rates of renal endpoints, such as albuminuria, increased serum creatinine, and the need for dialysis or transplantation [17, 18]. Intriguingly, these protective effects appear to be independent of the glucose‐lowering effects of SGLT2 inhibitors. While the exact mechanisms underlying these cardiorenal protective effects are still being investigated, strong evidence has led to the American Heart Association, the American College of Cardiology, and the Heart Failure Society of America (AHA/ACC/HFSA) releasing guidelines recommending SGLT2 inhibitors for patients with T2DM and cardiovascular or renal risk [19].

However, there is a risk of muscle loss associated with the use of SGLT2 inhibitors, especially in older adults or those with underlying muscle conditions [20, 21, 22]. This may be due to hormonal changes and increased protein breakdown [12]. Hence, careful patient monitoring is crucial. However, significant knowledge gaps remain regarding long‐term impacts on frailty and muscle mass, necessitating detailed body composition studies.

GLP‐1 receptor agonists (e.g., exenatide, liraglutide, and semaglutide) mimic the actions of the incretin hormone GLP‐1, which is released in response to food intake [23, 24]. By activating GLP‐1 receptors, these medications stimulate insulin secretion from pancreatic beta cells, suppress glucagon secretion from alpha cells, and slow gastric emptying [25]. These actions improve glycemic control, increase satiety, and reduce food intake, which together lead to weight loss [26]. GLP‐1 receptor agonist‐based therapies have emerged as powerful weight‐loss interventions, achieving outcomes comparable to surgical approaches [27]. However, their long‐term impact on muscle health requires further investigation.

SGLT2 inhibitors and GLP‐1 receptor agonists demonstrate varied impacts on body composition in T2DM management [12]. The composition of the human body is a complex physiological construct comprised of fat mass and fat‐free mass, with the latter encompassing bone and lean body mass. Lean body mass is subdivided into skeletal muscle mass, organ tissues, and total body water [28]. Pharmacological interventions targeting weight reduction, such as SGLT2 inhibitors and GLP‐1 receptor agonists, potentially modulate these compositional components; however, the specific mechanisms and effects on each component are not fully understood. A major concern, especially in older adults, is the unintended loss of skeletal muscle mass, which can have significant negative health consequences. Aging is a critical factor in the development of sarcopenia, a condition characterized by the progressive loss of skeletal muscle, strength, and function [29]. Sarcopenia is a significant complication in T2DM, with older adults at heightened risk [30]. Given that aging exacerbates vulnerability to muscle loss, SGLT2 inhibitor use in older adults requires careful consideration [29, 31].

T2DM and sarcopenia have a complex, bidirectional relationship. T2DM can accelerate muscle loss, while muscle deterioration may worsen T2DM through reduced myokine production, which is crucial for glucose and fat metabolism [32, 33]. In older adults with T2DM, several factors increase the risk of sarcopenia, including a low body mass index (BMI), a sedentary lifestyle, hypertension, neuropathy, and calcium deficiency [34]. The underlying mechanisms linking T2DM and sarcopenia involve multiple processes, including insulin resistance, chronic inflammation, advanced glycation end‐product accumulation, oxidative stress, and mitochondrial dysfunction [33]. These processes collectively impair protein metabolism and vascular function, ultimately compromising muscle health. Aging amplifies these detrimental effects, making older adults particularly susceptible to the dual burden of T2DM and sarcopenia. Based on this background information, we performed a meta‐analysis to evaluate the effects that SGLT2 inhibitors and GLP‐1 receptor agonists have on body weight, fat mass, and skeletal muscle mass, with a focus on determining whether these drugs contribute to sarcopenia in older adults.

## 2 Methods

### 2.1 Study Design

This study consisted of a systematic review and meta‐analysis that examined changes in body weight, muscle mass, and total fat mass in response to SGLT2 inhibitors in patients with T2DM. The study followed the Preferred Reporting Items for Systematic Reviews and Meta‐Analyses (PRISMA) guidelines and was registered in the International Prospective Register of Systematic Reviews (PROSPERO; registration number: CRD42024511420).

### 2.2 Search Strategy

A systematic literature search was performed in September 2024 across the PubMed, Embase, Scopus, and Cochrane databases for articles published between January 2005 and September 2024, with an updated search in December 2025 to identify newly published studies. The following keywords were used: (“GLP‐1 receptor agonist” OR “Semaglutide” OR “Liraglutide” OR “Dulaglutide” OR “Exenatide” OR “Albiglutide” OR “Lixisenatide” OR “SGLT2 inhibitor” OR “Canagliflozin” OR “Dapagliflozin” OR “Empagliflozin” OR “GIP”) AND “Diabetes mellitus” AND (“Muscle mass” OR “Fat‐free mass” OR “Lean mass”). The reference lists of the identified articles were also searched manually.

### 2.3 Study Selection

Articles that met the following criteria were included: original study available as a full‐text article; randomized controlled trial (RCT) that compared SGLT2 inhibitor treatment with either a placebo or other hypoglycemic medication; and the study reported comprehensive outcomes, including changes in body weight, muscle mass, and fat mass in T2DM patients. Duplicate articles, inaccessible articles, studies with incomplete outcome data, studies with a non‐RCT design, and studies that contained animal experiments were excluded. Three authors independently performed the article selection and screening. Articles were considered eligible when approved by at least two reviewers.

### 2.4 Data Extraction

The data extraction was performed independently by two reviewers (WJ and NP). The following data were extracted from each included study: first author's name, title, publication year, sample size, country, study design, population characteristics (age, gender, and health status), intervention and comparison characteristics (drug name, dose, duration of treatment, placebo, and control group), and outcome measures (changes in body weight, muscle mass, and fat mass).

### 2.5 Quality Assessment of Studies and Risk of Bias

Two authors (NB and SL) independently assessed the quality and risk of bias of the studies using the Critical Appraisal Skills Programme (CASP) checklist for RCTs. Each study was evaluated across all 11 domains of the checklist.

### 2.6 Statistical Analysis

Statistical analysis was performed using R software (version 4.4.1) with the metafor package [35]. The heterogeneity among the studies was assessed using Cochran's Q test and I^2^ statistics. The results were grouped by outcome of interest and are presented in both textual and table form. Publication bias was evaluated using funnel plots. When *p* < 0.01, the result was considered statistically significant. The statistical value of each outcome of interest was determined using standard deviation (SD) and standardized mean difference (SMD) values. When the SMD is 0.2, 0.5, or 0.8, the effect size is considered small, medium, or large, respectively [36, 37, 38].

## 3 Results

### 3.1 Article Selection

**FIGURE 1:**
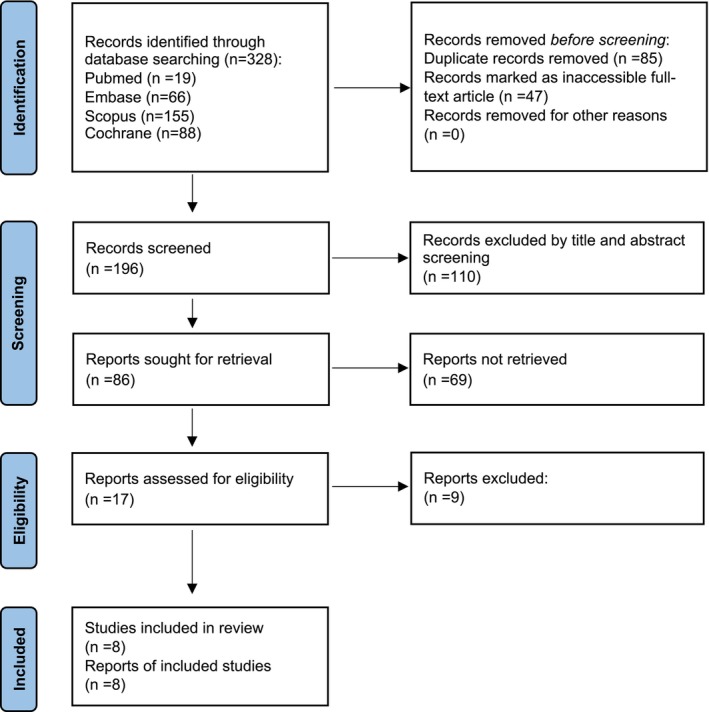
PRISMA flow diagram of study selection process.

Among the 328 articles identified in the comprehensive search, 85 were duplicate records and were subsequently removed. A further 47 articles were excluded due to the unavailability of the full text. Subsequently, 110 articles were excluded following title and abstract screening, while another 69 articles were removed due to insufficient data. Eight articles met the eligibility criteria and were retained for analysis. The selection process is shown in Figure 1. In addition, an updated search identified two recently published studies investigating the effects of GLP‐1‐based therapies on body weight, muscle mass, and total fat mass in T2DM patients, which are shown in Figure S1. These studies were analyzed separately rather than pooled with SGLT2 inhibitor studies due to differences in mechanisms of action and the limited number of available studies.

The diagram illustrates the systematic search and screening process for identifying eligible studies examining SGLT2 inhibitor effects on body composition in older adults with type 2 diabetes. Studies in supplementary box were identified in December 2025 update and analyzed separately (see Data S1).

### 3.2 Characteristics of the Articles and Quality Assessment

The characteristics of the eight included articles are shown in Table 1. Interestingly, all eight studies were conducted in Asian countries: Japan, Korea, and Taiwan. All studies were RCTs published between 2005 and 2024, and they investigated older patients diagnosed with T2DM and various underlying health conditions, including cardiovascular disease, nonalcoholic fatty liver disease, and kidney dysfunction. The age range of the participants was 52.5 ± 10.3 to 74.2 ± 4.9 years. Six of the eight articles reported a T2DM duration of approximately 8–15 years.

**TABLE 1:** Characteristics of included studies examining the effects of SGLT2 inhibitors on body composition in older adults with type 2 diabetes.

Author(s), publication year	Eligible criteria		Location	Group		Treatment group					CASP
	T2DM	Underlying conditions		Control	Treatment	Age (years)	Total	M	F	T2DM duration (years)	Total 11
Kayano et al. 2020	T2DM	Hypertension (grade 1 or 2) and/or a history of ischemic heart disease	Japan	Conventional	Dapagliflozin	69.4 ± 7.1	36	31	5	8.1 ± 4.7	11
Yamakage et al. 2020	T2DM	Patients who were receiving diet and exercise therapy	Japan	Conventional	Dapagliflozin	58.4 ± 13.0	27	11	16	N/A	11
Horibe et al. 2022	T2DM	Pateints who had estimated glomerular filtration rates of > 45 mL/min/1.73 m^2^	Japan	Conventional	Dapagliflozin	59.7 ± 12.0	26	16	10	11.8 ± 8.1	11
Zeng et al. 2022	T2DM	N/A	Taiwan	Linagliptin	Empagliflozin	58.9 ± 9.9	46	23	23	13.0 ± 6.1	9
Hoshika et al. 2022	T2DM	Patients with Acute Myocardial Infarction and T2DM	Japan	Placebo	Empagliflozin	67.5 ± 8.8	25	19	6	N/A	10
Yabe et al. 2023	T2DM	N/A	Japan	Placebo	Empagliflozin	74.2 ± 4.9	64	48	16	12.4 ± 8.2	11
Han et al. 2020	T2DM	Pateints who exhibited NAFLD	Korea	Metfomin + Pioglitazone	Ipragliflozin	52.5 ± 10.3	30	19	11	9.1 ± 6.0	11
Inoue et al. 2019	T2DM	Pateints who had estimated glomerular filtration rates of > 45 mL/min/1.73 m^2^	Japan	Conventional	Ipragliflozin	60.5 ± 9.8	24	13	11	15.9 ± 7.7	11

*Note:* All studies were randomized controlled trials (RCTs) conducted in Asian countries (Japan, Korea, Taiwan) and published between 2005 and 2024. Age is presented as mean ± standard deviation (SD). CASP scores range from 0 to 11, with higher scores indicating lower risk of bias.

Abbreviations: CASP, critical appraisal checklists; F, female; M, male; N/A, not applicable; NAFLD, nonalcoholic fatty liver disease; T2DM, type 2 diabetic mellitus.

Patients in the intervention groups received one of three SGLT2 inhibitor drugs: dapagliflozin (three studies), empagliflozin (three studies), or ipragliflozin (two studies). Patients in the control groups received conventional medication for T2DM, a placebo, linagliptin, or a combination of metformin and pioglitazone.

In terms of the quality of the included studies and the risk of bias in the studies, six studies achieved the highest possible CASP checklist score of 11, as shown in Table 1. The two remaining articles received scores of 9 and 10 out of 11, which are considered acceptable values in the bias assessment.

In addition, baseline characteristics of two GLP‐1 receptor agonist studies are shown in Supplementary Table 1. Both RCTs were recently published in 2025 and conducted in the USA and China, recruiting older adult T2DM patients aged 56.0 ± 9.8 and 54.74 ± 10.01 years, respectively [39, 40]. Treatment groups received either retatrutide (0.5, 4, 8, or 12 mg) or ebenatide (2 mg), while control groups received placebo or dulaglutide (1.5 mg). Both studies scored 11/11 on the CASP checklist assessment. Treatment groups received retatrutide or ebenatide, while controls received placebo or dulaglutide. Both studies scored 11/11 on the CASP checklist assessment. Coskun et al. [39] evaluated 0.5, 4, 8, and 12 mg retatrutide versus placebo or 1.5 mg dulaglutide in 103 T2DM patients over 36 weeks. Xu et al. [40] assessed 2 mg ebenatide versus placebo over 24 and 52 weeks.

### 3.3 Outcomes Assessment

This review focused on three primary outcomes: muscle mass, body weight/BMI, and fat mass (Table 2). Among the 272 T2DM patients who received SGLT2 inhibitors, 32.4% (*n* = 88) received dapagliflozin, 48.9% (*n* = 133) received empagliflozin, and 18.8% (*n* = 51) received ipragliflozin. Changes in each parameter from the baseline value were evaluated at week 24 of treatment, with one empagliflozin‐based study extending to week 52.

**TABLE 2:** Changes in body weight, muscle mass, and total fat mass with SGLT2 inhibitor treatment.

Author (s), publication year	Treatment group			Result (s)					
				Body weight (kg)/BMI (kg/m^2^)		Muscle mass (kg)		Total fat mass (kg)/cm^2^	
	(*n*)	Drug	Duration (weeks)	Change from baseline mean ± SD/95% CI/mean SE	(*p*‐value)	Change from baseline mean ± SD/95% CI/mean SE	(*p*‐value)	Change from baseline mean ± SD/95% CI/mean SE	(*p*‐value)
Kayano et al. 2020	36	Dapagliflozin	24	−1.0 ± 0.8	< 0.001	−0.3 ± 3.8	N/A	−1.9 ± 2.5	< 0.001
Yamakage et al. 2020	26	Dapagliflozin	24	−3.2 (−4.1, −2.4)	N/A	0.1 (−1.0, 1.2)	N/A	IFA −9.8 (−17.7, −2.0) SFA −16.3 (−31.2, −1.4)	N/A
Horibe et al. 2022	26	Dapagliflozin	24	−2.40 ± 2.13	N/A	−0.64 ± 1.38	N/A	−2.32 ± 1.95	N/A
Zeng et al. 2022	46	Empagliflozin	24	−1.55 (0.32)	< 0.001	−0.44 (0.36)	0.221	−1.02 (0.41)	0.016
Hoshika et al. 2022	22	Empagliflozin	24	−0.31 ± 0.26	≤ 0.05	−0.19 ± 0.24	N/A	−1.47 ± 0.14	0.15
Yabe et al. 2023	65	Empagliflozin	52	−3.27 ± 0.25	N/A	−1.57 ± 0.35	N/A	−1.77 ± 0.28	N/A
Han et al. 2020	29	Ipragliflozin	24	−1.6 ± 0.4	≤ 0.05	−0.8 ± 0.3	≤ 0.05	−1.0 ± 0.3	≤ 0.05
Inoue et al. 2019	22	Ipragliflozin	24	−2.78 ± 0.40	N/A	−0.56 ± 0.34	N/A	−2.07 ± 0.28	N/A

*Note:* Data are presented as mean ± SD, mean change with 95% CI, or mean ± SE from baseline to endpoint. *p*‐values represent statistical significance of within‐group changes from baseline or between‐group differences where indicated. Negative values indicate reduction from baseline. Treatment duration for all studies was 24 weeks except Yabe et al. (52 weeks).

Abbreviations: BMI, body mass index; CI, confidence interval; IFA, intraperitoneal fat area; N/A, not applicable; SD, standard deviation; SE, standard error; SFA, subcutaneous fat area.

The results of all the studies indicated that the SGLT2 inhibitors significantly reduced the body weight of the T2DM patients compared to the control treatments, while muscle mass was generally maintained. Dapagliflozin significantly reduced total fat mass, including intraperitoneal and subcutaneous fat. Ipragliflozin significantly reduced total fat mass, particularly visceral fat. The effect of empagliflozin on fat mass varied depending on the dosage and duration, with a tendency toward attenuated fat accumulation in the long term. Therefore, the SGLT2 inhibitors significantly promoted weight loss by reducing fat accumulation in the T2DM patients.

Table S2 shows body weight, muscle mass, and total fat mass outcomes from the two additional GLP‐1 studies. Coskun et al. evaluated multiple retatrutide doses in 103 T2DM patients over 36 weeks compared with placebo or dulaglutide. Xu et al. assessed ebenatide efficacy over 24 and 52 weeks compared with placebo. Retatrutide at 8 and 12 mg significantly reduced body weight, total fat mass, and muscle mass at 36 weeks (*p* < 0.0001), with 4 mg also effectively reducing body weight. Ebenatide at 2 mg promoted weight loss at both 24 and 52 weeks compared with placebo. Body fat decreased at 24 weeks with preserved muscle mass. However, muscle mass declined slightly by 52 weeks.

### 3.4 Meta‐Analysis of SGLT2 Inhibitors on Body Composition

#### 3.4.1 Effect of SGLT2 Inhibitors on Body Weight

**FIGURE 2:**
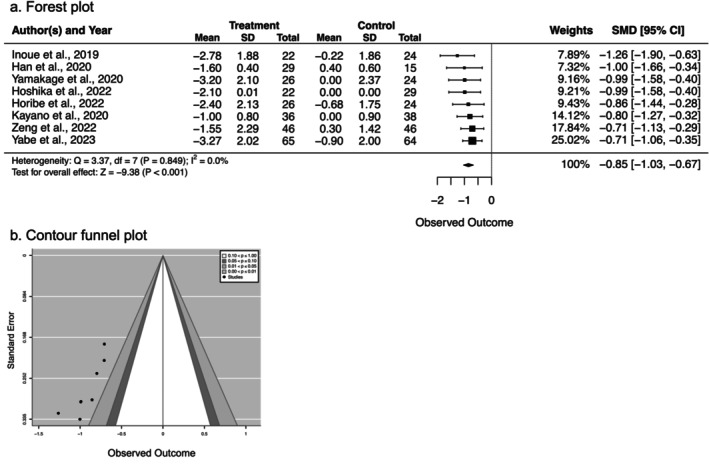
Effect of SGLT2 inhibitors on body weight/BMI in older adults with type 2 diabetes. (a) Forest plot showing standardized mean difference (SMD) with 95% confidence intervals (CI). Negative values favor SGLT2 inhibitor treatment. The diamond represents the pooled effect size. Heterogeneity was assessed using *I* ^2^ statistics. (b) Contour funnel plot for publication bias assessment, with contour lines representing statistical significance levels.

The meta‐analysis results showed that the patients who received an SGLT2 inhibitor (*n* = 272) demonstrated a significant reduction in body weight compared to those in the control groups (*n* = 269). The mean SD was 0.85, which is considered a large effect size (SMD = −0.85, 95% CI [−1.03, −0.67], *p* < 0.001) (Figure 2a). Notably, there was no heterogeneity among the studies (*I* ^2^ = 0.0%, *p* = 0.849), indicating that the SGLT2 inhibitors effectively reduced body weight in all eight studies. Therefore, the significance values of all the studies were plotted within the 0.00 < *p* ≤ 0.001 area of the contour funnel plot (Figure 2b). These results strongly indicate that SGLT2 inhibitors effectively reduce body weight and support their clinical application as treatments for T2DM patients with obesity.

#### 3.4.2 Effect of SGLT2 Inhibitors on Muscle Mass

**FIGURE 3:**
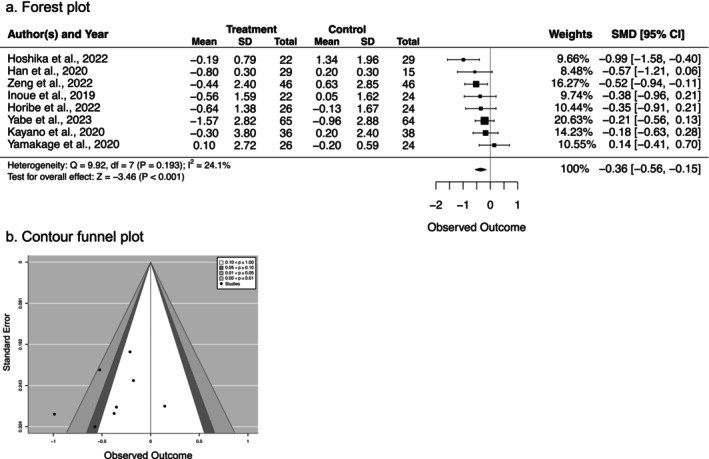
Effect of SGLT2 inhibitors on skeletal muscle mass in older adults with type 2 diabetes. (a) Forest plot showing SMD with 95% CI. Negative values indicate muscle mass reduction with SGLT2 inhibitor treatment. The diamond represents the pooled effect size. Heterogeneity was assessed using *I* ^2^ statistics. (b) Contour funnel plot for publication bias assessment.

When studies with similar designs, populations, and interventional drugs were assessed in this meta‐analysis, it was found that patients treated with SGLT2 inhibitors demonstrated a reduction in muscle mass compared to those in the control groups. The mean effect size of 0.36 SD indicated a small effect (SMD = −0.36, 95% CI [−0.56, −0.15], *p* < 0.001). Seven of the eight articles reported muscle mass loss in the patients who received an SGLT2 inhibitor, which indicated that there was low heterogeneity among the articles (*I* ^2^ = 24.1%, *p* = 0.193) (Figure 3a). Notably, the contour funnel plot revealed a borderline significant change in the muscle mass in the patients who received an SGLT2 inhibitor in five studies (0.1 < *p* ≤ 1.0), whereas significant reductions were observed in two studies (0.05 < *p* ≤ 0.1 and 0.01 < *p* ≤ 0.05, respectively) (Figure 3b).

Despite the statistical significance of the pooled effect, the small effect size and the finding that only two of eight studies showed significant individual reductions suggest that muscle mass changes are modest and may not be clinically meaningful for most patients. These results indicate that SGLT2 inhibitors have a measurable but small effect on muscle mass in older adults with T2DM, with the predominant weight loss occurring through fat mass reduction.

#### 3.4.3 Effect of SGLT2 Inhibitors on Fat Mass

**FIGURE 4:**
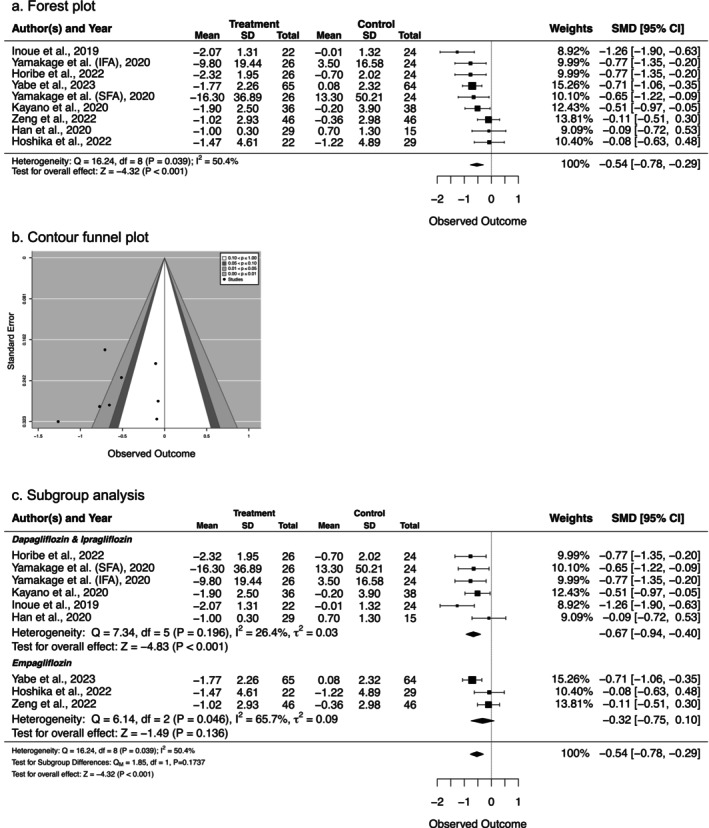
Effect of SGLT2 inhibitors on total fat mass in older adults with type 2 diabetes. (a) Forest plot showing overall effect. (b) Contour funnel plot for publication bias assessment. (c) Subgroup analysis by SGLT2 inhibitor type (dapagliflozin/ipragliflozin vs. empagliflozin). Data are presented as SMD with 95% CI. Negative values favor SGLT2 inhibitor treatment (indicating fat mass reduction). The diamonds represent the pooled effect sizes. Heterogeneity was assessed using *I* ^2^ statistics.

All eight studies evaluated the effect of SGLT2 inhibitors on fat mass, including total fat mass and intraperitoneal, subcutaneous, and visceral fat. As shown in Figure 4a, there was a significant reduction in fat mass in the treatment groups compared to the control groups, with a medium‐sized effect (SMD = −0.54, 95% CI [−0.78, −0.29], *p* < 0.001). These results suggest that SGLT2 inhibitors can reduce fat accumulation in T2DM patients with obesity. However, moderate heterogeneity was observed (*I* ^2^ = 50.4%, *p* = 0.039), as reflected in the broad distribution of the funnel plot (Figure 4b). Therefore, a subgroup analysis by drug type was performed to investigate the source of this heterogeneity.

Three SGLT2 inhibitors were tested in the eight studies: dapagliflozin (three studies), ipragliflozin (two studies), and empagliflozin (three studies). Dapagliflozin and ipragliflozin were administered for 24 weeks, while empagliflozin was administered for both 24 and 52 weeks. Subgroup analysis was performed for two groups: dapagliflozin and ipragliflozin (DI) and empagliflozin (EM). As shown in Figure 4c, a significant reduction in total fat mass was observed in both the DI group (SMD = −0.67, 95% CI [−0.94, −0.40], *p* < 0.001) and the EM group (SMD = −0.54, 95% CI [−0.78, −0.29], *p* < 0.001). Notably, the heterogeneity was lower in the DI group compared to the overall analysis (*I* ^2^ = 26.4%, *p* = 0.196), suggesting that dapagliflozin and ipragliflozin significantly reduced total fat accumulation. However, the persistent heterogeneity in the empagliflozin group (*I* ^2^ = 65.7%, *p* = 0.046) likely reflects differences in treatment duration (24 vs. 52 weeks) and dosing regimens across studies, as empagliflozin effects may be time‐dependent. These results suggest that SGLT2 inhibitors contribute to apparent clinical weight loss in T2DM patients by significantly reducing total fat accumulation with minimal impact on muscle mass.

### 3.5 Meta‐Analysis of GLP‐1‐Based Therapies on Body Composition

Following completion of the main SGLT2 inhibitor analysis, two recent GLP‐1‐based therapy studies were subsequently identified. These studies are present separately as supplementary analyses due to the limited number of studies and their publication after the original search period.

#### 3.5.1 Effect of GLP‐1‐Based Therapies on Body Weight

Meta‐analysis of ebenatide and retatrutide on body weight in T2DM patients (*n* = 155) is shown in Figure S2a. These agonists significantly promoted weight loss compared with placebo and dulaglutide, a GLP‐1 receptor agonist (*n* = 76), with a moderate‐to‐large effect (SMD = −0.76 [−1.09, −0.44], *p* < 0.001). However, heterogeneity was high (*I* ^2^ = 71.2%) due to minimal effect of the lowest retatrutide dose (0.5 mg). The contour funnel plot showed significance values distributed within the 0.1 < *p* ≤ 0.001 area (Figure S2b), indicating dose‐dependent responses. These results demonstrate significant weight reduction effects at appropriate doses.

#### 3.5.2 Effect of GLP‐1‐Based Therapies on Muscle Mass

Figure S3a shows the effect of these GLP‐1‐based agents on muscle mass using the same subjects and therapeutic agents. Meta‐analysis demonstrated that retatrutide at 4, 8, and 12 mg significantly decreased muscle mass, while 0.5 mg retatrutide and 2 mg ebenatide did not affect muscle mass. The pooled effect was moderate (SMD = −0.63 [−1.05, −0.22], *p* = 0.003) with high heterogeneity (*I* ^2^ = 82.1%). The contour funnel plot revealed significance values within the 0.1 < *p* ≤ 0.001 area (Figure S3b), indicating dose‐dependent variability. Collectively, higher doses of the tri‐agonist significantly reduced muscle mass, whereas lower doses and the GLP‐1 receptor agonist showed minimal effects.

#### 3.5.3 Effect of GLP‐1‐Based Therapies on Total Fat Mass

Body fat mass was significantly reduced with these therapies compared with placebo and GLP‐1 receptor agonist groups (Figure S4a). Meta‐analysis revealed significant fat reduction across various doses. The tri‐agonist versus dulaglutide comparison showed a large effect (SMD = −0.87 [−1.33, −0.40], *p* < 0.001), while the overall pooled effect was moderate (SMD = −0.69 [−1.04, −0.33], *p* < 0.001). Heterogeneity remained relatively high (*I* ^2^ = 75.2%), consistent with body weight and muscle mass analyses. Significance values were distributed within the 0.1 < *p* ≤ 0.001 area of the funnel plot (Figure S4b). Taken together, these therapies effectively reduce total fat mass in obese T2DM patients.

## 4 Discussion

In this meta‐analysis, we examined the effects of SGLT2 inhibitors on body composition, with a focus on their impact on fat and muscle mass. By systematically analyzing the effects of these antidiabetic medications on body weight, fat, and skeletal muscle, we sought to address growing concerns about the development and progression of sarcopenia in patients receiving these treatments. Notably, the eligible studies predominantly enrolled older adults, allowing us to gain valuable insights into the effects of these medications in this particularly vulnerable population at heightened risk for sarcopenia.

SGLT2 inhibitors are now indicated beyond T2DM for chronic kidney disease (CKD) and heart failure regardless of diabetes status [19, 41, 42, 43]. Our meta‐analysis focuses exclusively on patients with T2DM, representing only a portion of the current SGLT2 inhibitor users. The body composition effects observed in our diabetic cohort may not translate to nondiabetic populations, as T2DM‐specific metabolic alterations, including insulin resistance, hyperglycemia, and altered protein metabolism, may differentially influence muscle and fat responses. Nondiabetic patients with CKD or heart failure may have distinct nutritional states, inflammatory profiles, and muscle wasting mechanisms. Our findings therefore apply to older adults with T2DM, and extrapolation to nondiabetic populations requires caution and dedicated studies.

Our meta‐analysis included eight RCTs (a total of 541 T2DM patients) that compared the effects of SGLT2 inhibitors with those of conventional antidiabetic medications on body composition. All eight studies demonstrated significant body weight reduction, with six reporting substantial fat mass loss, which was consistently significant across the studies. Although the overall results indicated that there were significant reductions in both fat and muscle mass—in alignment with previous analyses—the pooled analysis showed that the observed muscle mass reduction was driven by two studies, as most of the studies did not show significant changes. These findings demonstrate that SGLT2 inhibitors favorably affect body composition primarily through fat mass reduction, though their impact on muscle mass requires careful interpretation.

This meta‐analysis demonstrated that SGLT2 inhibitors provide a favorable benefit–risk profile for body composition in patients with T2DM. Based on GRADE assessment (Table 3), high‐certainty evidence from eight studies involving 541 participants demonstrated that SGLT2 inhibitor therapy produced a large and consistent reduction in body weight or BMI (SMD −0.85, 95% CI −1.03 to −0.67), with no observed heterogeneity (*I* ^2^ = 0.0%). Moderate‐certainty evidence from the same studies showed that SGLT2 inhibitors significantly reduced total fat mass, including visceral fat, with a moderate effect size (SMD −0.54, 95% CI −0.78 to −0.29), although moderate heterogeneity was present (*I* ^2^ = 50.4%). In contrast, moderate‐certainty evidence from seven to eight studies involving 495 participants indicated that SGLT2 inhibitor use was associated with a small but consistent reduction in muscle mass (SMD −0.36, 95% CI −0.56 to −0.15), with low heterogeneity (*I* ^2^ = 24.1%). These effects were robust across studies, supporting the reliability of observed benefits. Although muscle mass loss was modest and substantially smaller than fat loss, this finding warrants clinical consideration, particularly in older patients or those at sarcopenia risk. The predominance of fat over muscle loss suggests favorable body recomposition overall. Nonetheless, monitoring muscle mass and implementing supportive strategies such as resistance exercise and adequate protein intake may further optimize the benefit–risk balance.

**TABLE 3:** GRADE evidence quality assessment for SGLT2 inhibitor effects on body composition.

Outcome	No. of participants (studies)	Effect (SMD, 95% CI)	Magnitude of effect	Heterogeneity (*I* ^2^)	Certainty of evidence (GRADE)	Interpretation
Body weight/BMI (benefit)	541 (8 studies)	−0.85 [−1.03, −0.67]	Large	0.0%	High	Consistent and clinically meaningful weight reduction
Total fat mass (benefit)	541 (8 studies)	−0.54 [−0.78, −0.29]	Moderate	50.4%	Moderate	Significant reduction in total and visceral fat
Muscle mass (risk)	495 (7–8 studies)	−0.36 [−0.56, −0.15]	Small	24.1%	Moderate	Small reduction in muscle mass; clinical significance uncertain

*Note:* Data are presented as SMD with 95% CI and the number of participants and studies per outcome. Effect magnitude was classified according to GRADE guidance, considering effect size, confidence interval precision, and between‐study heterogeneity (I^2^).

Abbreviations: BMI, body mass index; CI, confidence interval; GRADE, Grading of Recommendations, Assessment, Development and Evaluations; SMD, standardized mean difference.

Regarding the effect on fat, empagliflozin showed time‐dependent effects, with significant reductions observed only in the 52‐week treatment group, while shorter treatment durations showed no significant effects [44, 45]. However, the subgroup analysis based on different SGLT2 inhibitor types revealed significant fat mass reductions across the tested drug categories.

In terms of the effect on muscle mass, only Zeng et al. [46] reported a reduction in muscle mass when they compared SGLT2 inhibitors to linagliptin (a dipeptidyl peptidase 4 [DPP‐4] inhibitor). This suggests that SGLT2 inhibitors may have a greater impact on muscle mass than DPP‐4 inhibitors. In contrast, in their study on Japanese T2DM patients [47], found that DPP‐4 inhibitors, such as sitagliptin, may protect against muscle loss by reducing fat mass without affecting fat‐free mass. This benefit could be due to reduced inflammation [48, 49] and preserved GLP‐1 activity [50, 51]. However, another study found that teneligliptin did not impact the muscle mass of hemodialysis patients [52]. Overall, inhibiting DPP‐4 seems to mediate a protective or neutral effect on muscle health; hence, DPP‐4 inhibitors are potentially safe for use in elderly T2DM patients with sarcopenia [53].

Our results show that the current research findings in this area are inconsistent. Some studies indicate lean body mass and muscle loss [31, 54], while others suggest no significant effect or even an increase in lean body mass [12, 55], which encompasses muscle, organs, and body water [28]. Compared to traditional glucose‐lowering therapies, SGLT2 inhibitors promote greater body water loss by increasing glucose excretion, and the loss of water results in weight reduction [11, 56]. Studies using bioimpedance spectroscopy have shown that SGLT2 inhibitor‐related weight loss primarily results from the loss of adipose tissue and a transient reduction in extracellular fluid, which is linked to the activation of the renin‐angiotensin‐aldosterone system [57]. Therefore, SGLT2 inhibitor‐related weight reduction is likely primarily attributable to fat mass and body water loss and to minimally impact muscle mass.

Although rare cases of sarcopenia and myopathy have been reported with the use of SGLT2 inhibitors, particularly in older individuals [20, 21], recent evidence suggests that these inhibitors have muscle‐protective effects. For example, in diabetic mice, luseogliflozin prevented muscle atrophy, increased muscle weight and grip strength, improved fatty acid profiles, and reduced the levels of key molecules involved in fat metabolism and muscle breakdown, such as Forkhead box protein O1 [58, 59]. These promising preclinical results with luseogliflozin justify further clinical investigation of its muscle‐related effects in diabetic patients. In humans, empagliflozin has been shown to effectively reduce fat mass and visceral adipose tissue, leading to weight loss [55]. In another study, a slight decrease in skeletal muscle mass was observed; however, the overall muscle‐to‐fat ratio improved, and no significant changes in sarcopenia, sarcopenic obesity, fat‐free mass, or muscle strength were noted [60]. Empagliflozin has also been found to improve insulin sensitivity, reduce hepatic steatosis, and decrease liver enzymes [55].

These conflicting findings highlight the need for further research to clarify the clinical effects of SGLT2 inhibitors on muscle health, particularly in older adults. While the evidence for SGLT2 inhibitor‐induced muscle loss is inconclusive, potential mechanisms that have been proposed include the calorie deficit induced by increased urinary glucose excretion contributing to muscle breakdown for energy in patients with preexisting sarcopenia, if the deficit is not compensated for by dietary adjustments [7, 61]. Additionally, glycosuria‐induced osmotic diuresis can lead to dehydration, which can potentially prevent muscle protein synthesis [62]. Finally, while SGLT2 inhibitors can indirectly lower insulin and raise glucagon levels, potentially affecting glucose and amino acid uptake in the liver and muscles, these effects are not typically observed in clinical practice [63, 64]. Specifically, the risks of exacerbated hypoglycemia in patients who restrict carbohydrates and accelerated muscle loss are not typically seen. However, high glucagon levels promote lipolysis, which can lead to ketone production and, in rare cases, ketosis or ketoacidosis [65]. Given these complex and contradictory findings with SGLT2 inhibitors, understanding how alternative antidiabetic therapies affect body composition is equally important.

This study initially aimed to evaluate the effects of both SGLT2 inhibitors and GLP‐1 receptor agonists on fat and muscle mass. However, a comprehensive literature search revealed a significant insufficiency of RCTs investigating the long‐term effects of GLP‐1 receptor agonists on body composition that met our PICO criteria. Consequently, a robust meta‐analysis of GLP‐1 receptor agonist effects could not be performed. While these agonists show promise for weight loss, the current literature lacks sufficient long‐term data regarding their impact on muscle mass and frailty. Current evidence suggests a potential adaptive response, but factors like age and preexisting muscle weakness may influence individual outcomes [66]. Therefore, further research, particularly detailed body composition studies, is crucial to clarify the effects of both GLP‐1 receptor agonists and SGLT2 inhibitors on fat and muscle composition and to ensure patient safety.

The incretin‐based therapy landscape has expanded beyond traditional GLP‐1 receptor agonists to include dual and triple agonists. Tirzepatide, a dual GLP‐1/GIP receptor agonist, has emerged as a particularly important therapeutic agent, demonstrating substantial weight loss in clinical trials [67]. Additional agents include ebenatide, a long‐acting GLP‐1 receptor agonist, and retatrutide, a triple GLP‐1/GIP/glucagon agonist, achieving significant weight loss [39, 40]. These agents are increasingly prescribed for both T2DM and obesity management at varying dosing regimens depending on the therapeutic indication [68, 69, 70].

Mechanistically, SGLT2 inhibitors and incretin‐based therapies differ fundamentally. SGLT2 inhibitors promote weight loss primarily through renal glucosuria‐mediated caloric deficit and osmotic diuresis, while GLP‐1 receptor agonists act via central appetite suppression, enhanced satiety signaling, and delayed gastric emptying. Dual and triple agonists provide additional mechanisms. GIP coactivation in tirzepatide enhances adipose tissue metabolism and lipid distribution, while glucagon coactivation in retatrutide increases energy expenditure and promotes lipolysis [71, 72, 73, 74]. These differential mechanisms raise important questions about whether specific receptor engagement profiles differentially influence the muscle‐to‐fat ratio of weight loss. While emerging evidence suggests potential adaptive responses with relative muscle preservation, the magnitude and clinical significance vary based on patient‐specific factors and the agent used.

Emerging clinical data from dual and triple agonists provide insights into these differential body composition effects. Tirzepatide demonstrates substantial weight reduction in phase 3 trials, with MRI‐based body composition analyses indicating reductions in muscle volume that appear adaptive to the degree of weight loss, while improving muscle fat infiltration beyond expected weight‐related changes [75, 76]. Retatrutide demonstrates greater weight loss with proportional reductions in both fat and muscle mass at higher doses, while ebenatide achieves moderate weight loss with preserved muscle mass during short‐term treatment but gradual muscle reduction with prolonged use (Figure S2) [39, 40]. These patterns suggest that the degree of muscle preservation varies based on receptor engagement profile, dosing regimen, and treatment duration. However, dedicated long‐term studies examining muscle strength, physical function, and frailty outcomes in older adults remain limited.

Despite widespread use in older populations, long‐term data on muscle mass, strength, functional capacity, and frailty remain insufficient. Direct head‐to‐head comparative studies between SGLT2 inhibitors and incretin‐based therapies examining body composition in older adults are critically needed to guide treatment selection in elderly patients at risk of sarcopenia [75, 76, 77].

The limitations of our study that may have impacted our results include the small sample sizes in some of the included RCTs and the limited number of studies that met our PICO criteria. Furthermore, the exclusive focus on Asian populations limits the generalizability of our findings to other demographics.

Body composition differences across ethnic groups remain debated, with some evidence suggesting that Asian populations exhibit higher body fat and lower skeletal muscle mass at comparable BMI than other ethnic groups, although findings are inconsistent when adjusted for age and BMI [78, 79, 80]. Our analysis included exclusively Asian populations, which limits generalizability to other ethnicities, despite the use of ethnicity‐specific BMI thresholds for Asian groups. Ethnic variations in dietary patterns, genetic polymorphisms, physical activity behaviors, and drug metabolism may influence baseline sarcopenia risk as well as treatment responses. Therefore, the effect sizes observed in our Asian cohort may not directly translate to other populations, and validation studies in Western and other ethnic groups are necessary before broader generalization.

While we addressed the high degree of heterogeneity among the studies through a subgroup analysis of different SGLT2 inhibitor types, other factors, such as variations in the methods used to assess body composition and treatment duration, should also be considered when interpreting our results. Importantly, a strength of this study is that we focused on the effects of SGLT2 inhibitors in older adults, who are at heightened risk of sarcopenia, which can significantly impact their quality of life and functional independence. Our findings highlight the need for more dedicated studies on this demographic.

To address the gaps in our knowledge about the effects of SGLT2 inhibitors on muscle health, clinical trials must be conducted to assess the long‐term effects of SGLT2 inhibitors on muscle mass, strength, and function in diverse populations, especially older adults and those with preexisting sarcopenia. Further research should explore combining SGLT2 inhibitors with exercise and nutritional interventions to optimize muscle preservation. Comparative studies of different SGLT2 inhibitors and other antidiabetic drugs (e.g., DPP‐4 inhibitors and GLP‐1 receptor agonists) will help clarify whether the effects these medications have on muscle are agent‐specific or class‐wide. Conducting dedicated trials in vulnerable populations, including elderly and frail patients and those with comorbidities, is essential for the development of personalized treatment protocols. Critically, future research should investigate (1) the molecular mechanisms by which SGLT2 inhibitors affect muscle metabolism (e.g., muscle growth, atrophy, regeneration, and repair) and muscle stem cells, (2) the influence of patient‐specific factors, and (3) the differential impact of SGLT2 inhibitors on muscle fiber types. The use of reliable and standardized tools and methodologies (e.g., measurement techniques, biomarkers, and study designs) is crucial for translating preclinical findings and developing personalized treatments that optimize glycemic control and preserve muscle integrity in populations at risk of sarcopenia.

From a clinical perspective, sarcopenia can significantly impair the physical functioning and psychosocial well‐being of elderly diabetic patients. However, early intervention, including lifestyle modification, exercise, and nutrition, offers promising prevention opportunities. With its focus on older adults, our meta‐analysis underscores the importance of conducting further research to better understand the effects of SGLT2 inhibitors on muscle health in this group and to develop strategies that mitigate the risk of sarcopenia while optimizing metabolic outcomes.

In conclusion, SGLT2 inhibitors demonstrate a favorable benefit–risk profile for body composition in patients with T2DM, combined with established cardiovascular and renal benefits, supporting continued use in older adults. Weight reduction occurs predominantly through fat mass loss, and the associated muscle mass reduction is modest. Our supplementary analysis of GLP‐1‐based therapies suggests similar weight loss profiles across antidiabetic drug classes, though muscle effects may vary by specific agent and dose. We recommend monitoring muscle mass during treatment, particularly in individuals at risk of sarcopenia.

## 5 Conclusion

Current findings on SGLT2 inhibitor effects on muscle health show variable results across studies. While our meta‐analysis demonstrated a statistically significant reduction in muscle mass, the small effect size indicates modest changes that may not be clinically meaningful for most patients. Rare case reports of sarcopenia or myopathy in older individuals raise some concern, though these appear to be exceptional cases. Importantly, our findings demonstrate that SGLT2 inhibitor‐related weight loss occurs predominantly through fat mass reduction rather than muscle loss, suggesting a favorable body composition profile overall. Future research should include well‐designed clinical trials with longer follow‐up periods to better characterize muscle health outcomes, particularly in vulnerable populations such as frail older adults or those with preexisting sarcopenia. Studies should aim to identify at‐risk populations, optimize combination therapies with exercise and nutrition, elucidate underlying mechanisms, and develop personalized treatment strategies. Based on current evidence, clinicians should monitor muscle mass in older adults receiving SGLT2 inhibitors, particularly those with preexisting muscle‐related conditions or frailty. However, for the majority of older adults with T2DM, SGLT2 inhibitors appear to offer favorable metabolic and cardiovascular benefits with weight loss occurring predominantly through fat mass reduction. The small observed changes in muscle mass should be weighed against the substantial proven benefits of SGLT2 inhibitors in this population.

## Author Contributions

W.J., N.B., S.L., and N.P. conceived and designed the study. W.J., N.B., P.S., S.L., and N.P. performed the literature search, data extraction, and study quality assessment. N.K. performed the statistical analyses and prepared the tables and figures. W.J. and N.P. drafted the manuscript. N.B. and S.L. reviewed and edited the manuscript. All authors read and approved the final manuscript.

## Funding

The authors have nothing to report.

## Conflicts of Interest

The authors declare no conflicts of interest.

## Supporting information


**Data S1:** prp270232‐sup‐0001‐Supinfo.docx.

## Data Availability

The data are available from the corresponding author upon reasonable request. The protocol was registered in PROSPERO (CRD42024511420).
