# Supplementary material for: The Effects of SGLT2 Inhibitors on Muscle Health in Older Adults: A Systematic Review and Meta‐Analysis
Source: Pharmacol Res Perspect. 2026 Mar 11;14(2):e70232. doi: 10.1002/prp2.70232 (PMC13140511; doi:10.1002/prp2.70232)
Supplement: Supplementary file 1 — Data S1: prp270232‐sup‐0001‐Supinfo.docx. [file PRP2-14-e70232-s001.docx]

**Supplementary Materials**

**
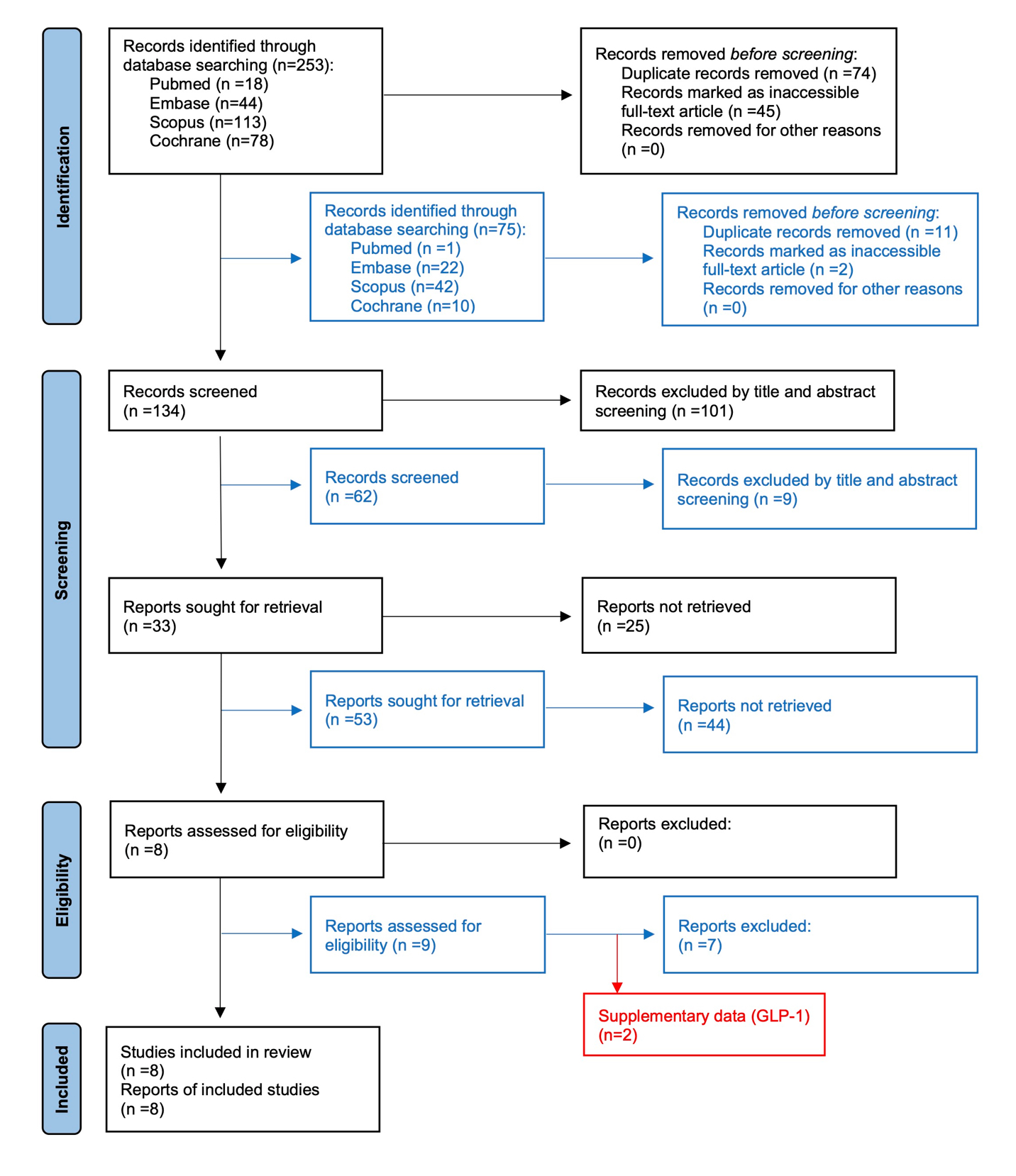
**

**Supplementary Figure 1. Updated PRISMA flow diagram for GLP-1-based therapy studies.**

The updated search identified two recently published GLP-1-based therapy studies examining body composition effects in older adults with type 2 diabetes that were published after the completion of the original systematic search.

**
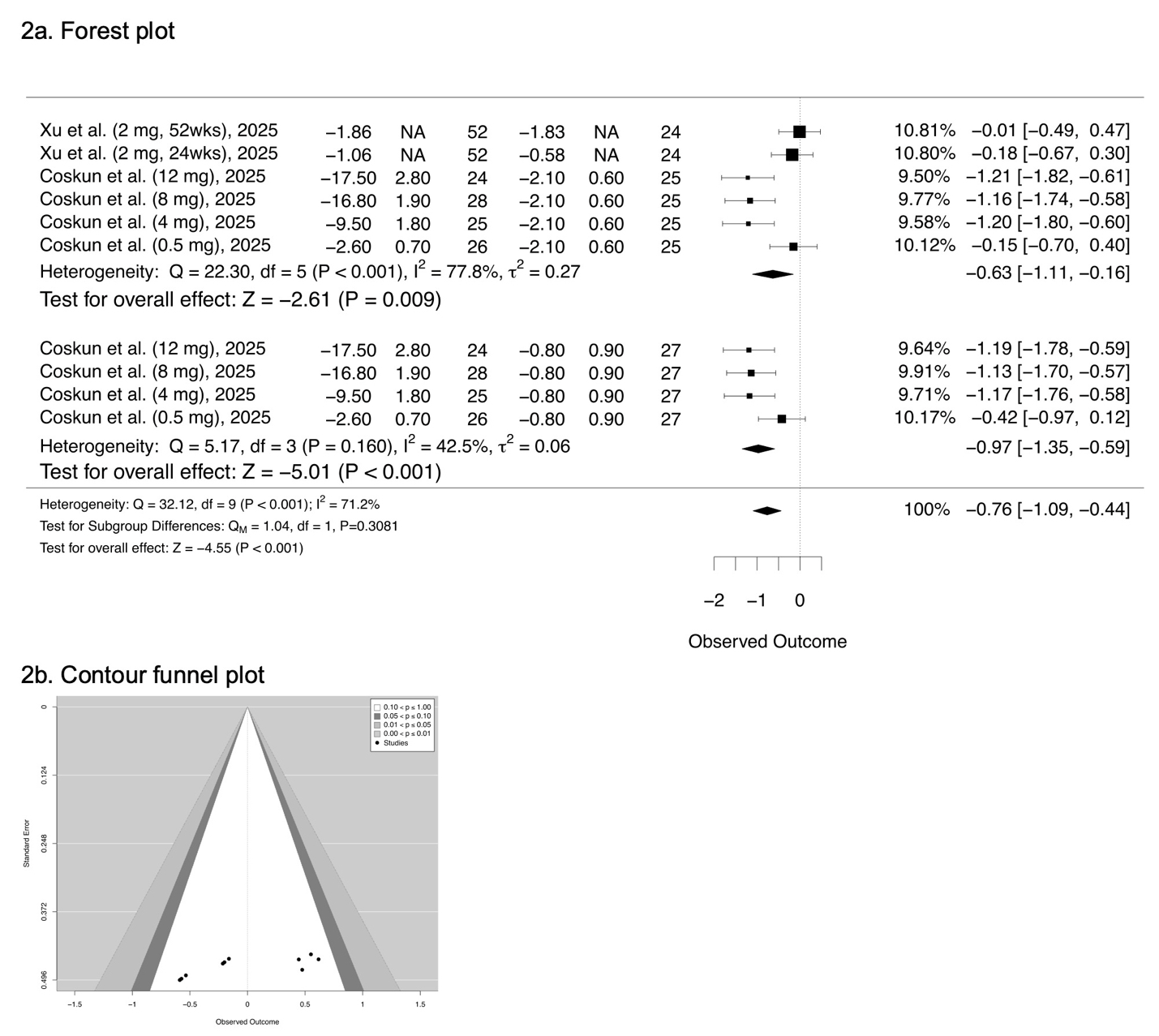
**

**Supplementary Figure 2. Effect of GLP-1-based therapies on body weight or body weight/BMI in older adults with type 2 diabetes.**

(a) Forest plot showing SMD with 95% CI for retatrutide (multiple doses) and ebenatide compared with placebo or dulaglutide. Negative values indicate weight/BMI reduction. The diamond represents the pooled effect size. Heterogeneity was assessed using I² statistics. (b) Contour funnel plot for publication bias assessment, with contour lines representing statistical significance levels.

**
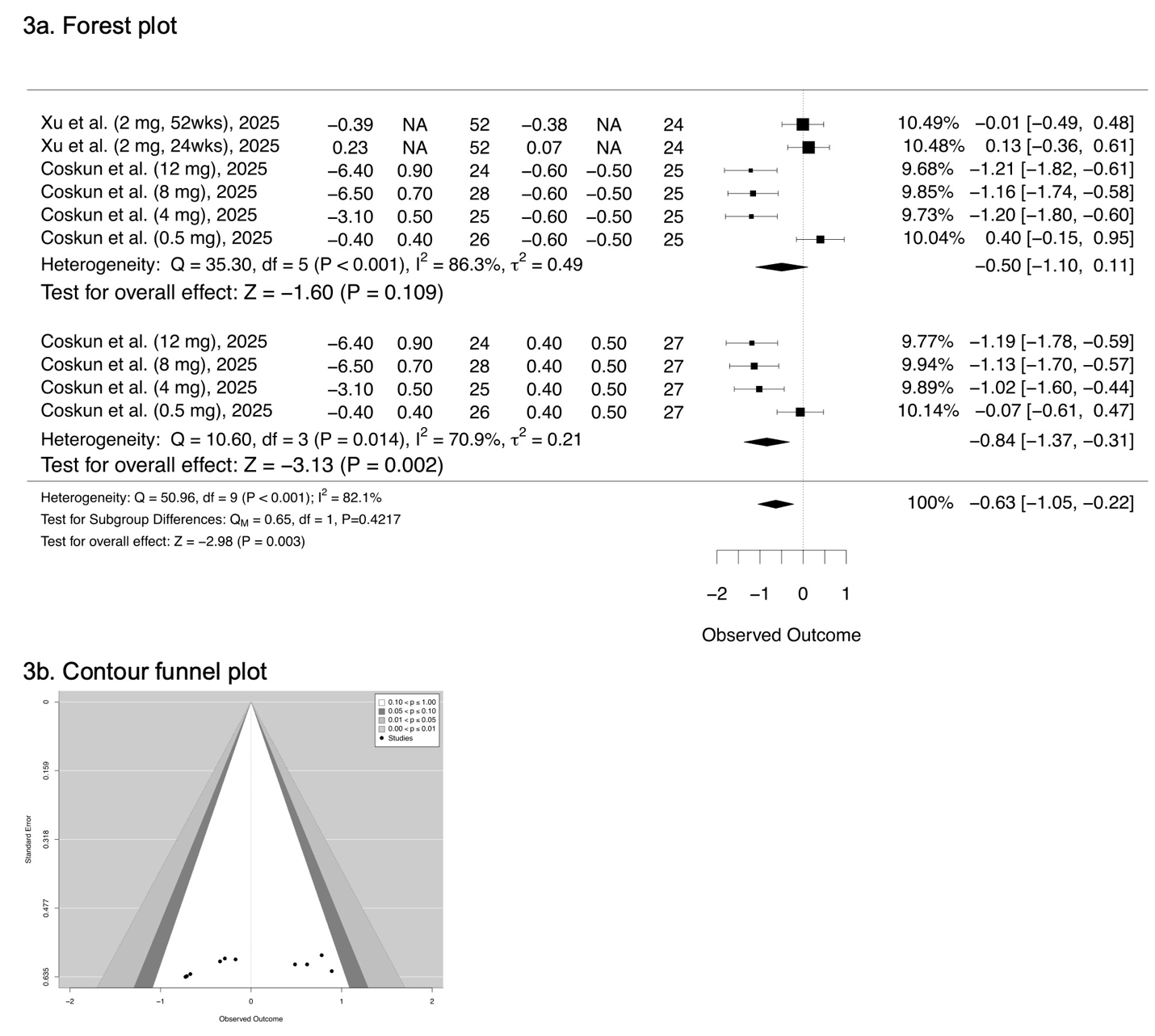
**

**Supplementary Figure 3. Effect of GLP-1-based therapies on skeletal muscle mass in older adults with type 2 diabetes.**

(a) Forest plot showing SMD with 95% CI for retatrutide (multiple doses) and ebenatide compared with placebo or dulaglutide. Negative values indicate muscle mass reduction. The diamond represents the pooled effect size. (b) Contour funnel plot for publication bias assessment.

**
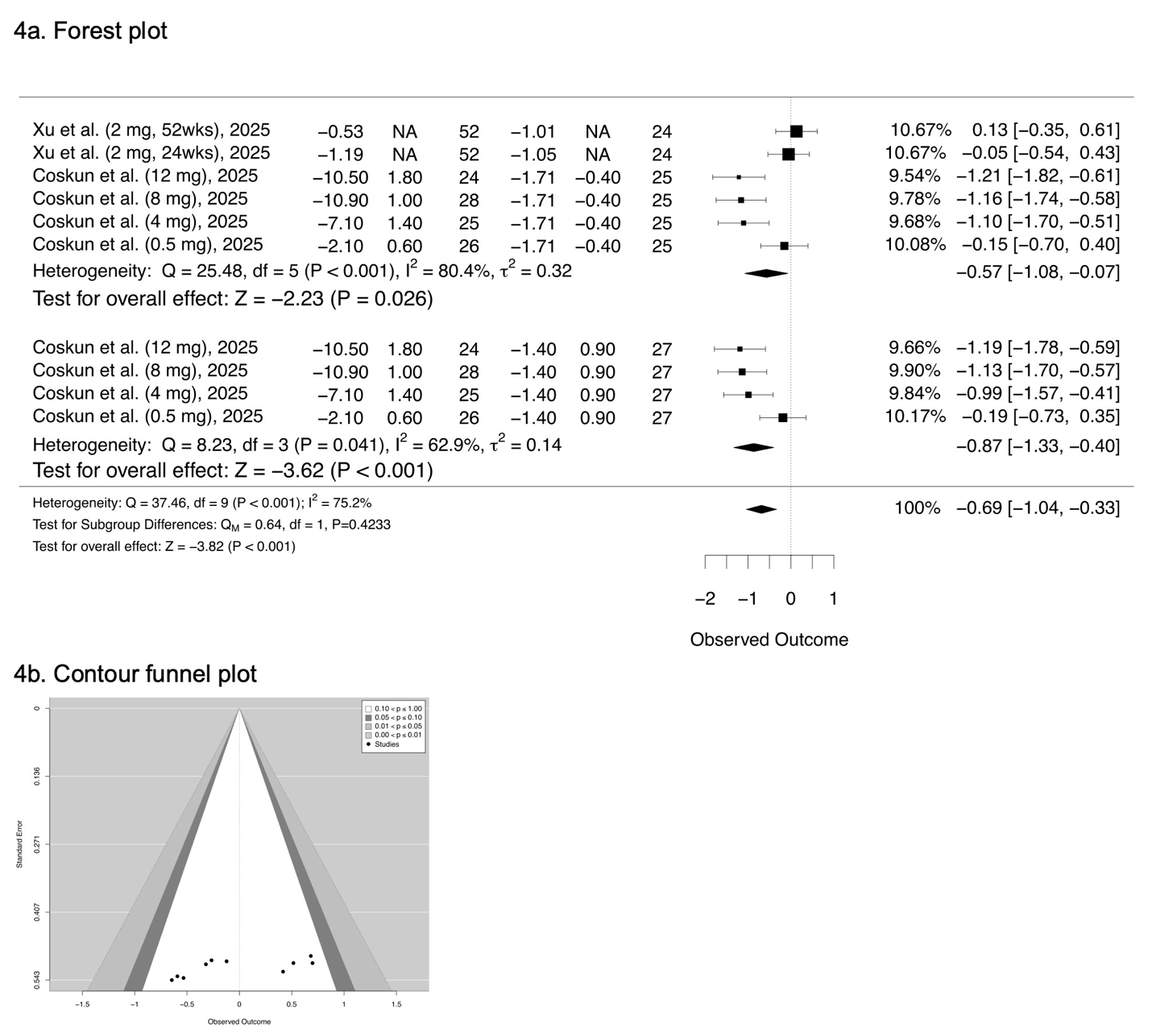
**

**Supplementary Figure 4. Effect of GLP-1-based therapies on total fat mass in older adults with type 2 diabetes.**

(a) Forest plot showing SMD with 95% CI for retatrutide (multiple doses) and ebenatide compared with placebo or dulaglutide. (b) Contour funnel plot for publication bias assessment. Data are presented as SMD with 95% CI. Negative values favor SGLT2 inhibitor treatment indicate fat mass reduction. The diamonds represent the pooled effect sizes.

**Supplementary Table 1. Characteristics of included studies examining the effects of GLP-1-based therapies on body composition in older adults with type 2 diabetes.**

| Author(s), | Eligible criteria | | Location | Group | | Treatment group | | | | | | | CASP | |  |
| --- | --- | --- | --- | --- | --- | --- | --- | --- | --- | --- | --- | --- | --- | --- | --- |
| Publication year | T2DM | Underlying conditions |  | Control | Treatment | Age (years) | Total | M | F | | T2DM duration (years) | | Total 11 | |  |
| Coskun et al. 2025 | T2DM | N/A | USA | Placebo  Dulaglutide | Retatrutide | 56.0 ± 9.8 | 189 | 84 | | 105 | | N/A | | 11 | |
| Xu et al. 2025 | T2DM | N/A | China | Placebo | Ebenatide | 54.74 ± 10.01 | 76 | 55 | | 21 | | N/A | | 11 | |
|  |  |  |  |  |  |  |  |  |  | |  | |  | |  |

T2DM, Type 2 diabetic mellitus; CASP, Critical appraisal checklists; M, Male; F, Female; N/A, not applicable. Both studies were randomized controlled trials (RCTs) identified through an updated systematic search and published in 2025. Age is presented as mean ± SD. Retatrutide is a triple GLP-1/GIP/glucagon receptor agonist; ebenatide is a long-acting GLP-1 receptor agonist. CASP scores range from 0 to 11, with higher scores indicating lower risk of bias.

**Supplementary Table 2. Changes in body weight, muscle mass, and total fat mass with GLP-1-based therapies.**

| Author (s), | Treatment group | | | Result (s) | | | | | |
| --- | --- | --- | --- | --- | --- | --- | --- | --- | --- |
| Publication |  | | | Body weight (kg) / BMI (kg/m^2^) | | Muscle mass (kg) | | Total fat mass (kg) / cm^2^ | |
| year | (n) | Drug and dose | Duration (weeks) | Change from baseline mean ± SD / 95% CI / mean SE | (*p* - value) | Change from baseline mean ± SD / 95% CI / mean SE | (*p* - value) | Change from baseline mean ± SD / 95% CI / mean SE | (*p* - value) |
| Coskun et al. 2025 | 26 | Retatrutide^a^  0.5 mg | 36 | −0.5 (−2.4, 1.4) | p = 0.59 | 0.2 (−1.2, −1.5) | p = 0.81 | −0.4 (−1.9, 1.0) | p = 0.59 |
| Coskun et al. 2025 | 25 | Retatrutide^a^  4 mg | 36 | −7.4 (−11.2, −3.7) | p < 0.0001 | −2.5 (4.0, −1.1) | p = 0.0006 | −5.4 (−8.3, −2.65) | p = 0.0003 |
| Coskun et al. 2025 | 28 | Retatrutide^a^  8 mg | 36 | −14.7 (−18.6, −10.9) | p < 0.0001 | −5.9 (−7.6, −4.3) | p < 0.0001 | −9.2 (−11.4, −7) | p < 0.0001 |
| Coskun et al. 2025 | 24 | Retatrutide^a^  12 mg | 36 | −15.4 (−21.0, −9.8) | p < 0.0001 | −5.8 (−7.8, −3.8) | p < 0.0001 | −8.8 (−12.6, −5) | p < 0.0001 |
| Coskun et al. 2025 | 26 | Retatrutide^b^  0.5 mg | 36 | −1.8 (−4.1, 0.5) | p = 0.13 | −0.9 (−2.1, 0.3) | p = 0.16 | −0.7 (−2.8, 1.4) | p = 0.50 |
| Coskun et al. 2025 | 25 | Retatrutide^b^  4 mg | 36 | −8.7 (−12.7, −4.7) | p < 0.0001 | −3.6 (−4.9, −2.3) | p < 0.0001 | −5.7 (−9, −2.4) | p = 0.0008 |
| Coskun et al. 2025 | 28 | Retatrutide^b^  8 mg | 36 | −16 (−20.2, −11.8) | p < 0.0001 | −7 (−8.5, −5.4) | p < 0.0001 | −9.5 (−12.1, −6.9) | p < 0.0001 |
| Coskun et al. 2025 | 24 | Retatrutide^b^  12 mg | 36 | −16.7 (−22.6, −10.7) | p < 0.0001 | −6.9 (−8.8, −4.9) | p < 0.0001 | −9.1 (−13.2, −5) | p < 0.0001 |
| Xu et al. 2025 | 52 | Ebenatide  2 mg | 24 | ﻿−0.48 | p = 0.464 | 0.16 | p = 0.608 | ﻿−0.14 | p = 0.83 |
| Xu et al. 2025 | 52 | Ebenatide  2mg | 52 | ﻿−0.03 | p = 0.968 | ﻿−0.01 | p = 0.981 | 0.48 | p = 0.602 |

BMI, Body mass index; SD, standard deviation; SE, standard error; CI, confidence interval. Data are presented as mean change with 95% CI from baseline to endpoint. P-values represent statistical significance of within-group changes from baseline. Negative values indicate reduction from baseline. Retatrutide^a^: compared with placebo group; Retatrutide^b^ : compared with Dulaglutide (1.5 mg) group. Coskun et al. evaluated multiple retatrutide doses (0.5, 4, 8, and 12 mg) over 36 weeks. Xu et al. evaluated ebenatide (2 mg) over 24 and 52 weeks.
